# Racial Disparities and Comorbidities: Network Analysis of Maternal Outcomes in Alabama

**DOI:** 10.1007/s40615-025-02440-7

**Published:** 2025-04-29

**Authors:** Yasin Fatemi, Haneen Ali, Jingyi Zheng

**Affiliations:** 1 Department of Industrial and Systems Engineering, Auburn University, Auburn, AL USA; 2 Health Services Administration Program, Auburn University, 351 W Thach Concourse, 7080 Haley Center, Auburn, AL 36849 USA; 3 Department of Mathematics and Statistics, Auburn University, Auburn, AL USA; 4 The Department of Mechanical and Industrial Engineering, Applied Science Private University, Amman, Jordan

**Keywords:** Race, Comorbidity, Network analysis, LBW, Disparity

## Abstract

**Introduction:**

The study employed a robust network analysis methodology to assess the effects of race and comorbidities on birth outcomes, using a dataset of 443,902 mothers in Alabama from 2014 to 2021.

**Methods:**

Four multimorbidity networks corresponding to White, Black, Asian, and American Indian and Alaska Native groups were constructed to explore distinct comorbidity patterns. The nodes in these networks represented various diseases, while the edges, quantified by the Salton Cosine Index, depicted the associations between these conditions. Additionally, two separate networks were analyzed for low birth weight (LBW) and normal birth weight (NBW) to identify the differential impacts of specific diseases. Feature selection methods including random forest and logistic regression were applied to pinpoint crucial intersections between the LBW and NBW networks, enhancing the granularity of the analysis.

**Results:**

The findings indicated significant racial disparities in the density of comorbidity networks, with more complex disease interactions observed among Black, American Indian and Alaska Native, and Asian groups compared to Whites. Preexisting hypertension and eclampsia emerged as significant risk factors for LBW in White and Black groups, while gestational hypertension was prevalent across multiple racial groups. The LBW network displayed greater density than the NBW network, highlighting the intricate connections between comorbidities leading to adverse birth outcomes.

**Conclusion:**

These insights underline the necessity for healthcare interventions tailored to the distinct health profiles of each racial group to effectively address and reduce maternal health disparities.

## Introduction

Maternal birth outcomes are critical indicators of both individual and public health, yet despite advancements in technology and medical informatics, persistent disparities exist among different racial groups. For instance, according to Centers for Disease Control and Prevention, the maternal mortality rate for Black women is 2.6 times greater than that for White women [1], underscoring significant racial disparities. Previous studies also reported disparities in the prevalence of specific comorbidities, such as gestational diabetes and hypertension, among different racial groups [2, 3]. Maternal health disparities among racial groups are influenced by a combination of structural racism [4], economic and social factors [5], healthcare access inequities [6], cultural barriers [7], and biased healthcare practices [8]. While genetic factors may contribute to individual health risks, race itself is a social construct that serves as a proxy for systemic disparities rather than a biological determinant of health outcomes [9–11].

Crucially, chronic diseases, such as hypertension and diabetes, have been shown to significantly impact maternal health outcomes. These conditions can lead to complications during pregnancy and childbirth, affecting both the mother and the child. However, understanding the full impact of these chronic conditions on maternal health outcomes requires an examination of the complex interplay between various diseases, particularly in the context of racial disparities.

The two terms used interchangeably in the current study for the existence of more than one disease in a patient are comorbidity and multimorbidity. Comorbidity can be defined as the presence of an index disease with an additional diagnosis [12]. Multimorbidity is defined as the presence of multiple chronic or acute diseases and medical conditions in one person [13].

The prevalence, causes, and consequences of multimorbidity during pregnancy and its impact on maternal and neonatal outcomes remain poorly understood [14–16]. Several recent studies have shown that comorbidity and multimorbidity during pregnancy are of paramount importance, as these concurrent health conditions can have profound implications for both maternal and fetal health [17–19]. Pregnancy inherently places physiological stress on the body. When combined with additional diseases, the body may find it challenging to manage both pregnancy and illness, leading to potential complications. Moreover, when multiple health issues coexist, they can compound these risks, necessitating specialized care and potentially affecting fetal outcomes [18, 20].

A review of the literature reveals a focus on singular health outcomes or traditional epidemiological approaches, leaving a gap in comprehensive, systemic analyses [17, 18, 21–24]. However, there remains a lack of research employing network analysis to understand the complex interplay of comorbidity and multimorbidity across racial lines in maternal health.

The present study utilizes network analysis to model multimorbidities [19], a method that examines relationships in a network, focusing on the links and attributes of the entities involved [25]. Network analysis is versatile and applicable to diverse systems, such as social, transportation, and biological networks [26]. In the health sector, it is used to analyze complex interactions between health factors, diseases, and social determinants of health, offering insights into disease co-occurrence and potential underlying conditions [27]. This approach, which is distinct from traditional statistical methods, aids in discerning patterns in comorbidity and disease connections within health networks.

The field of health-related network analysis encompasses a range of studies focusing on different diseases and demographic factors. McInerney et al. explored the relationship between mental health issues and diabetes [22]. Lee and Park studied obesity-related comorbidities in older adults [21]. Zhou et al. analyzed comorbidity patterns in ischemic heart disease, with a focus on sex and age differences [24]. Kalgotra and Sharda investigated gender disparities in multimorbidity [28], while Chami et al. focused on comorbidities in a low-income Ugandan setting [29]. Alhasoun et al. examined health conditions and demographics in Brazil [30]. Finally, Kalgotra and Sharda analyzed multimorbidity across racial and ethnic groups in the USA [31]. These studies collectively enhance our understanding of the complex interconnections of health conditions across diverse populations. Although the studies mentioned provide valuable insights into the impact of comorbidities on general health outcomes, there remains a gap in understanding how interactions between multiple comorbid conditions affect maternal health outcomes, especially considering racial disparities. This overview highlights the necessity for further research into how comorbidity interactions influence birth outcomes across different races. In this study, LBW is used as an indicator of pregnancy outcomes and health disparities among racial groups. According to the CDC, LBW results from intrauterine growth restriction, prematurity, or both and is associated with adverse health outcomes, including higher neonatal and fetal mortality, impaired growth, cognitive delays, and an increased risk of non-communicable diseases (NCDs) later in life. Infants with LBW are approximately 20 times more likely to die than those with NBW, highlighting its significance as a critical public health concern [32].

The present paper has three primary objectives:

1. The first aim of the current study was to investigate the impact of race on LBW by constructing four distinct networks for four racial groups—White, Black, Asian, and American Indian and Alaskan Native (AI/AN)—each with various diseases—to compare racial differences.
2. The second objective of the current study was to explore the influence of various diseases on LBW. To achieve this goal, the current study will construct two distinct networks for LBW and NBW to illustrate the differences between them.
3. The third aim of the current study was to investigate the interaction effects of race and disease on LBW using logistic regression and random forest, both machine learning techniques. However, we focused our analysis on the presence of one or two diseases because of the low number of patients with more than two diseases.

In this study, we analyze birth records from the Alabama Department of Public Health (ADPH) to examine the role of multimorbidity in birth outcomes. By constructing disease networks and leveraging machine learning models, we investigate how race and comorbidities interact to influence LBW. Our approach combines network metrics with predictive modeling to uncover significant patterns, which are discussed in detail to highlight their implications for maternal health.

## Methods

### Description Data Source and Study Design

This study is a retrospective cohort study utilizing birth records from mothers in Alabama to examine the relationship between maternal comorbidities, race, and LBW. First, network analysis is applied to model and analyze comorbidity patterns across different racial groups, identifying key disease interactions associated with LBW. Subsequently, machine learning techniques are employed to determine the most influential predictors of LBW, including maternal race, preexisting conditions, and their interactions. These data were provided by the Alabama Department of Public Health. The department specifically disclaims responsibility for any analyses, interpretations, or conclusions [33]. This center is responsible for collecting and tabulating health-related statistical data and operating the vital records system for the State of Alabama. This includes the preparation of various statistical analyses of natality, pregnancy, general mortality, infant mortality, causes of death, marriage, divorce, and other demographic and health-related data for the state. They collect, manage, and structure health data from vital records and health surveys. Additionally, they integrate this data with information from external sources, including the US Census and various national surveys and reports [33].The present study used data from mothers in Alabama who gave birth between 2014 and 2021, totaling 457,619 observations, which were reduced to 443,902 after applying the inclusion and exclusion criteria and cleaning the data. The inclusion and exclusion criteria were as follows:

1. Residency in Alabama
2. The participants belonged to the White, Black, Asian, AI/AN racial groups. Other racial groups were excluded from the analysis because of an insufficient number of patients, hence limiting the generalizability of the results.
3. Patients for whom more than one race was reported were excluded.
4. Presence of missing values in race and birth weight categories.

During data cleaning, yearly datasets were combined to form a unique set. Patients whose race was not specified or who belonged to other racial groups were excluded, as were patients whose data were missing. After cleaning the data, the dataset comprised 294,134 records for White patients, 140,864 for Black patients, 7433 for Asian patients, and 1471 for patients identified as AI/AN. This resulted in a cumulative total of 443,902 patient records.

### Measures

#### Outcome Variable

LBW is defined as a birth weight less than 2500 g (5.5 pounds) regardless of gestational age [32].

#### Predictor Variables

##### Morbidities

The dataset from ADPH includes a limited set of maternal comorbidities: preexisting hypertension, gestational hypertension, diabetes, obesity, hepatitis B/C, sexually transmitted infections (chlamydia, syphilis, gonorrhea, HIV), tocolysis, and eclampsia. These were the only diseases reported in the dataset and were therefore the only conditions considered in this study. Notably, the dataset does not use the International Classification of Diseases, 10 th Revision (ICD- 10), which may limit the inclusion of other relevant maternal health conditions.

##### Demographic Variables

The average age of a mother at the time of giving birth, the average number of distinct diagnoses per patient, the percentage of patients with private insurance, the percentage of patients with Medicaid, the percentage of mothers married at birth, the average number of parental visits during pregnancy per patient, and the percentage of patients with no morbidities.

##### Race-Morbidity Interaction Terms

Diseases × Races

### Multimorbidity (Comorbidity) Network Construction and Edge Threshold Selection

The present study’s approach to multimorbidity, considering the lifetime medical history of patients, is advantageous compared with the traditional definition that focuses only on diseases present at the time of examination. Using lifetime medical history provides a more comprehensive understanding of a patient’s health, capturing the chronic nature and progression of diseases. This method acknowledges that past health issues can have lasting impacts and contribute to current health status. Moreover, it aligns with the perspective that health is a cumulative life course experience, as suggested in various health studies [34, 35]. This approach allows for a more subtle understanding of disease interactions and their long-term effects on health.

A multimorbidity network developed from a patient contains a set of nodes connected through the edges. In this network, each node represents a specific reported disease associated with maternal health, either during or before pregnancy. The diseases are selectively sourced from our database and cover a range of conditions. In addition, the size of each node is designed to reflect the prevalence of the disease it represents, with larger nodes indicating a higher prevalence in certain networks or racial groups. This aspect of visualization allows for an in-depth understanding of the varying impacts of these conditions across different populations, underscoring the critical role of demographic-specific data in addressing maternal health challenges.

In our multimorbidity network, an important characteristic is the depiction of disease relationships through undirected edges. For example, the comorbidity between obesity and diabetes is shown as an undirected edge connecting the nodes of these diseases, intentionally avoiding any implication of a causal relationship. This non-directional approach is uniformly applied across the network, highlighting disease co-occurrence rather than causality.

Another critical feature of this network is how we visualize connections between diseases. Here, the thickness of each edge is the strength of the association. It is designed to represent either the strength of the association between two diseases or the number of patients with those comorbid conditions. A thicker edge, such as one connecting the nodes for diabetes and hypertension, suggests a stronger association or a greater number of patients diagnosed with both conditions in our dataset.

Traditionally, Pearson’s correlation coefficient has been used to model associations between diagnoses [36, 37], but its effectiveness is limited by sample size, reducing its capacity to detect less common comorbidities. To address this, we employed the Salton Cosine Index (SCI), which is less influenced by the overall number of observations [38] and focuses on the relative prevalence of disease pairs. The SCI is calculated using the following formula:

1 $${\mathrm{SCI}}_{ij}=\frac{{c}_{ij}}{\sqrt{{c}_{i}*{c}_{j}}}$$

where ${c}_{ij}$ is the co-occurrence of diseases *i* and *j*, and ${c}_{i}$ and ${c}_{j}$ are the individual prevalence of these diseases.

The statistical significance of the SCI was determined by evaluating its correlation. This methodology aligns with previous recommendations found in the literature [28, 31, 39] for establishing an SCI threshold. Initially, the present study focused on identifying the quantity of significantly correlated comorbidities within a network, framed by Pearson’s correlation coefficient. Subsequently, this number was compared with that in a network constructed using the SCI. A specific cutoff point was pinpointed where the number of significantly correlated comorbidities matched in both networks.

In the comprehensive database network analyzed using Pearson’s correlation coefficient, with a significance level set at 0.01, 43 out of 78 possible morbidity pairings showed a significant correlation. In parallel, at an SCI cutoff value of 0.0135, the number of comorbidities was 43. This led to the adoption of 0.0135 as the SCI threshold for generating various networks tailored to different racial groups. The subsequent phase involved contrasting these networks using certain network measures, which are outlined in more detail in the following section.

### Network Metrics

The structural properties of a network can be measured using several network metrics, including degree, density, closeness, and betweenness centrality [40].

#### Community Detection (Clustering)

We used the Louvain method, a heuristic tool for community discovery, to identify diseases that co-occur and possibly share common underlying risk factors. By measuring the density of connections inside disease clusters relative to connections between distinct clusters, this approach aims to maximize modularity [41].

#### Degree

As a basic but essential metric in network analysis, degree centrality quantifies the importance of a node by measuring the number of edges connected to the node. The degree centrality of a disease (node) in a multimorbidity network indicates how many direct connections it has with other diseases. In an undirected network, the degree centrality of a node is calculated as [40]:

2 $${C}_{D}(i)= \frac{\sum_{j=1}^{N}{X}_{ij}}{N-1}$$

where:

*C*_*D*_(*i*): is the degree of centrality.
*X*_*ij*_: is an element of the adjacency matrix, indicating the presence (1) or absence (0) of an edge between node *i* and node *j*.
*N*: is the total number of nodes in the network.

#### Density

The density of a network refers to the ratio of the actual number of edges in the network to the maximum number of possible edges. Density in a multimorbidity network indicates how common comorbidities are in a given population and indicates the potential complexity of managing patient health. This indicates the degree to which the network is nearly a full graph, in which each node is connected to every other node [42, 43].

3 $$D= \frac{E}{{~}^{N(N-1)}\!\left/ \!{~}_{2}\right.}$$

where:

*E*: is the total number of edges in the network.
*N*: is the total number of nodes in the network.
${~}^{N(N-1)}\!\left/ \!{~}_{2}\right.$: gives the maximum number of edges that an undirected graph can have without loops.

#### Closeness

In network analysis, closeness centrality is a metric that expresses a node’s degree of proximity to every other node in the network [40]. It displays the mean distance taken by the shortest path connecting each node in the graph to every other node. Because these nodes have the shortest links to all other nodes, this measure can help discover which nodes are best positioned to affect the whole network immediately. The average distance of a disease from other diseases in the network is determined by its closeness centrality. A disease with greater closeness has a greater risk of being diagnosed with other diseases in fewer steps.

The formula for calculating closeness ${C}_{C}$(v) centrality for node *v* in a connected graph is as follows:

4 $${C}_{C}\left(v\right)=\frac{N-1}{{\sum }_{u\ne v}d(u,v)}$$

where:

*d* (*u*, *v*): is the shortest path between nodes *v* and *u*.
*N*: is the total number of nodes in the network.
${\sum }_{u\ne v}d(u,v)$: is the sum of the shortest-path distances from node *v* to all other nodes *u* in the network.

#### Betweenness

Based on a node’s ability to act as a connector or bridge between various network nodes, betweenness centrality measures a node’s importance inside a network. It calculates how frequently a node bridges the shortest path between two other nodes. In a multimorbidity network, a disease with higher betweenness forms more bridges with other diseases [44]. The betweenness centrality formula for node *v* is as follows:

5 $${C}_{B}(\mathrm{v}) ={\sum }_{s\ne v\ne \mathrm{t}}\frac{{\sigma }_{st}(v)}{{\sigma }_{st}}$$

## Results

### Descriptive Statistics

The summary statistics of the dataset are presented in Table 1. To analyze differences between four groups, the Kruskal–Wallis test was used for numerical variables, and the chi-square test was applied for categorical variables. The *p* values for all variables were statistically significant (*p* < 0.001). This dataset represents medical records from Alabama covering hospital visits of mothers between 2014 and 2021. **Age**: The average age of the Asian mothers at birth was 31.24 years, followed by White (27.65 years), AI/AN (26.83 years), and African American Black (26.45 years) mothers. **The number of distinct diagnoses per patient**: The average number of distinct diagnoses per patient was highest among Blacks (0.72) and lowest among Asians. **For insurance**, 4980 (67%) of the Asian mothers had private insurance, which was the highest among the groups, while the percentage of Blacks was the lowest at 35,216 (25%). Blacks also had the highest Medicaid coverage at 98,605 (70%), compared with Whites 117,654 (40%), Asians 1858 (25%), and AI/AN 868 (59%). **Marital status**: Only 30,990 (22%) of Black mothers were married at birth, the lowest among all groups, whereas 6541 (88%) of Asian mothers were married. **The number of visits**: The average number of prenatal visits was greatest among Whites (11.10), followed by Asians (10.89), AI/AN (10.82), and Blacks (10.47). In addition, analysis of the distribution of comorbidities revealed that Asian patients had the highest proportion of patients with no comorbidities 5649 (76%), suggesting a lower prevalence of concurrent health issues among this group. Conversely, Black patients had a greater incidence of one comorbidity than did patients of other races 56,346 (40%). The rarity of more than two comorbidities across all racial categories, at approximately 2%, aligns with the focus of the current study on individuals with one or two health conditions.

**Table 1:** Summary statistics

Variables	White	Black	Asian	American Indian or Alaska Native	*p* value
No. of patients	294,134	140,864	7433	1471	NA
The average age of mothers at the time of giving birth (years)	(*M* = 27.65, SD = 5.52)	(*M* = 26.45, SD = 5.67)	(*M* = 31.24, SD = 4.92)	(*M* = 26.83, SD = 5.92)	< 0.001*
Average no. of distinct diagnoses per patient	(*M* = 0.50, SD = 0.71)	(*M* = 0.72, SD = 0.79)	(*M* = 0.30, SD = 0.59)	(*M* = 0.62, SD = 0.78)	< 0.001*
Average no. of parental visits during pregnancy per patient	(*M* = 11.10, SD = 6.14)	(*M* = 10.47, SD = 6.46)	(*M* = 10.89, SD = 4.32)	(*M* = 10.82, SD = 7.15)	< 0.001*
Percentage of patients with private insurance	158,832 (54%)	35,216 (25%)	4980 (67%)	515 (35%)	< 0.001*
Percentage of patients with Medicaid	117, 654 (40%)	98,605(70%)	1858 (25%)	868 (59%)	< 0.001*
Percentage of mothers married at birth	200,011 (68%)	30,990 (22%)	6541 (88%)	736 (50%)	< 0.001*
Percentage of patients with no. morbidities • 0 • 1 • 2 • > 2	179,422 (61%) 88,240 (30%) 23,53 (8%) 2941 (1%)	64,093 (45.5%) 56,346 (40%) 17,608 (12.5%) 2818 (2%)	5649 (76%) 1487 (20%) 297 (4%) 0 (0.0%)	780 (53%) 500 (34%) 147.2(10%) 44 (3%)	< 0.001*

*p value < 0.05

### Network Analysis of Different Racial Groups

**Fig. 1:**
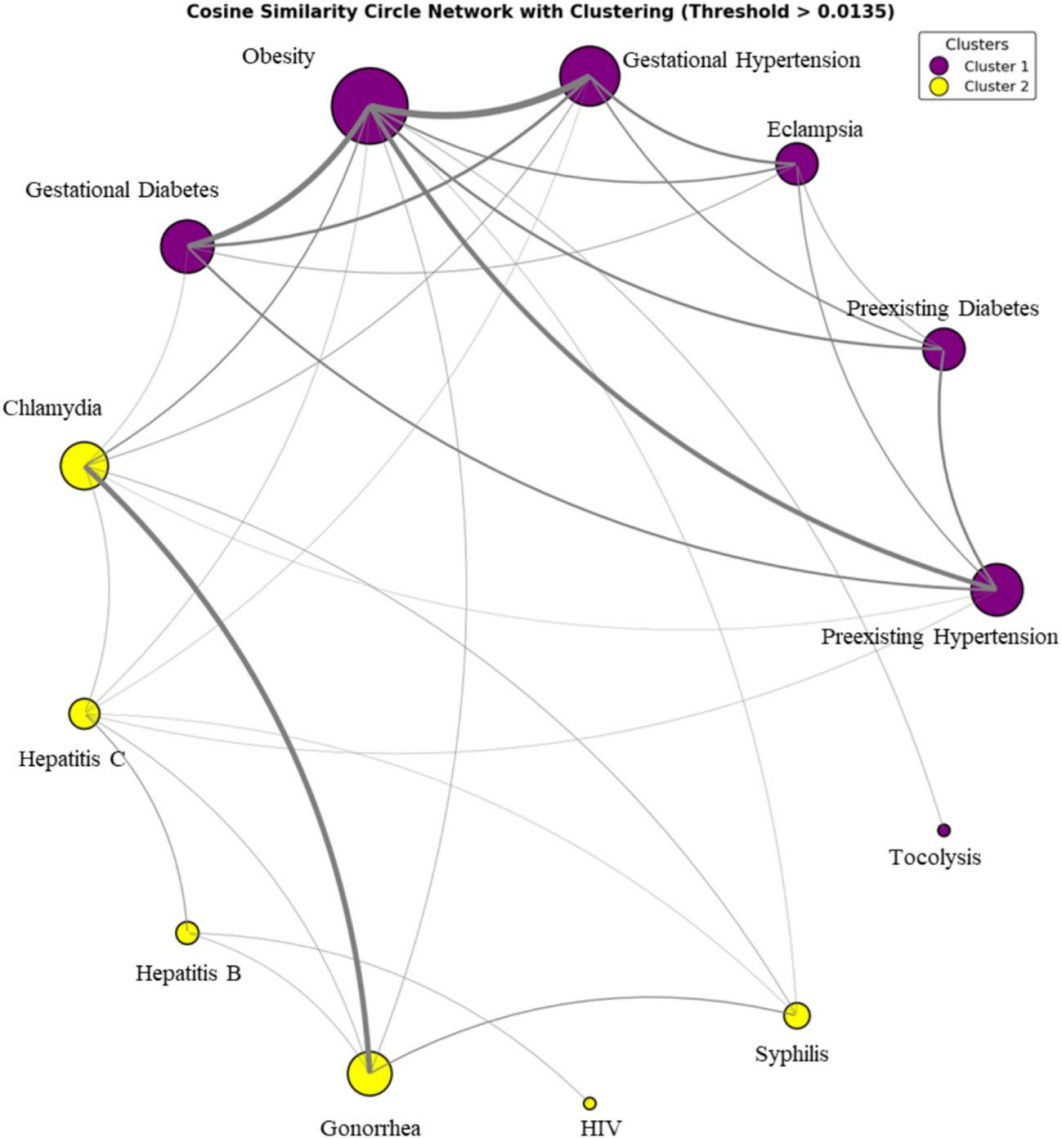
Comorbidity network for the White racial group

**Fig. 2:**
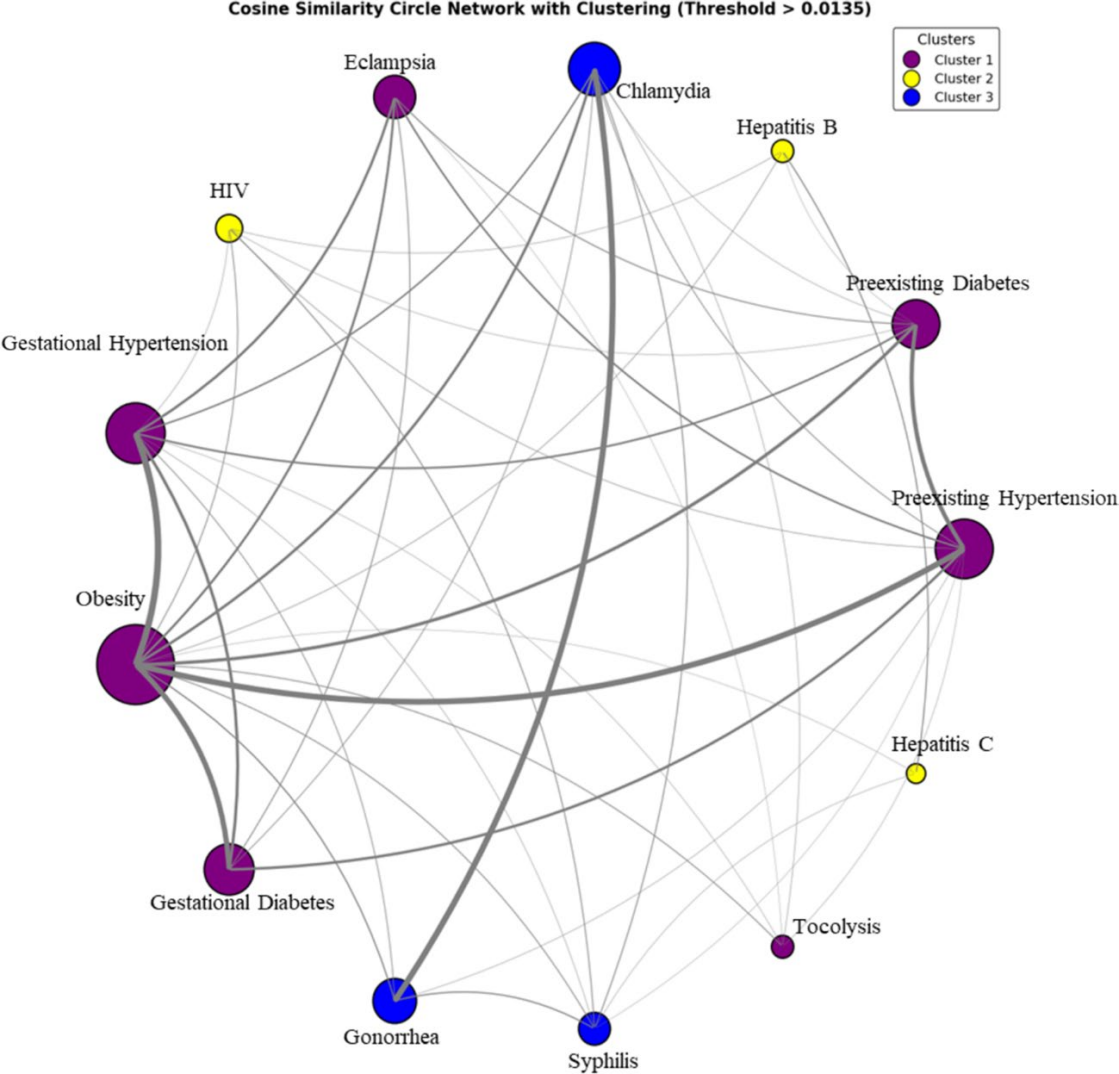
Comorbidity network for the Black racial group

**Fig. 3:**
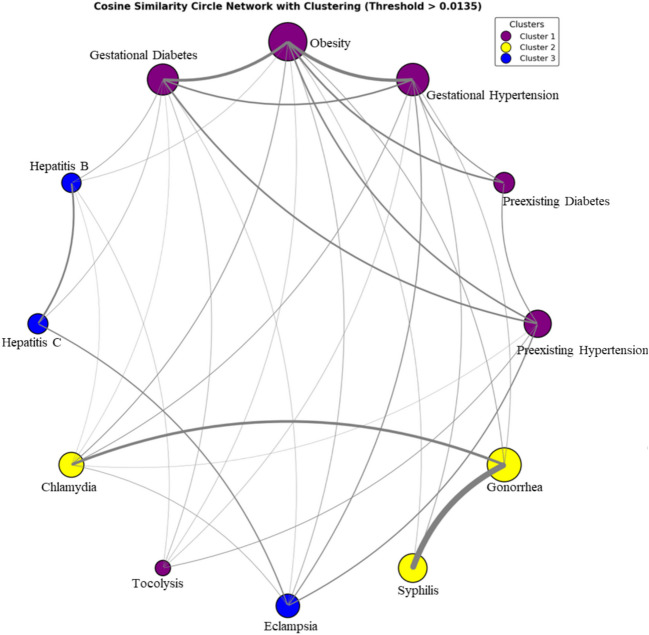
Comorbidity network for the Asian racial group

**Fig. 4:**
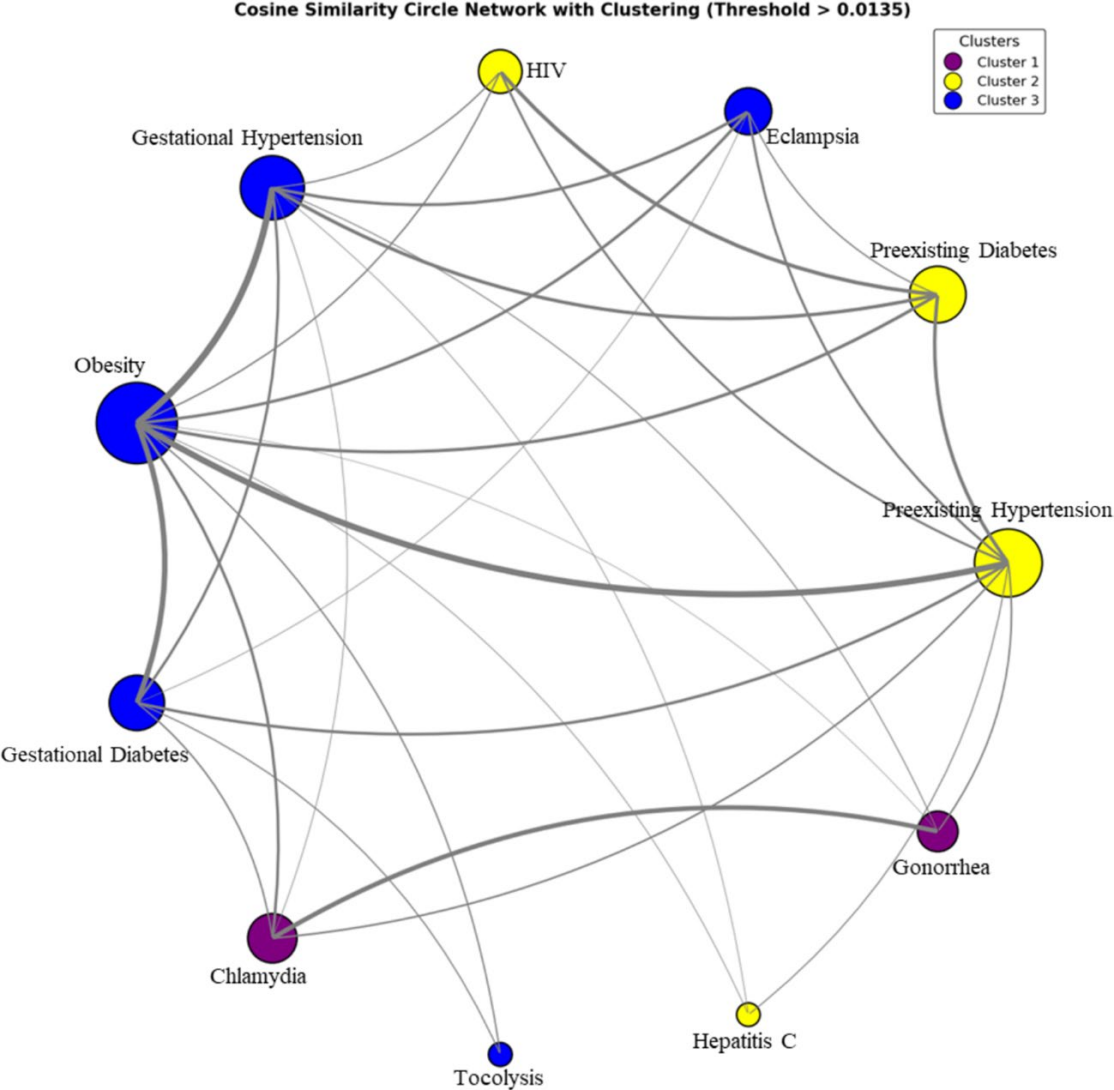
Comorbidity network for the AI/AN racial group

The present study tested the hypothesis that racial groups exhibit disparate outcomes in LBW, as detailed in Table 2. The chi-square test, shown in Table 2, demonstrated significant differences in LBW outcomes among various racial groups (*p* value < 0.001). To investigate disparities in the presence of comorbidities among different racial groups, we employed a racial network analysis. The findings from this analysis are presented in Figs. 1, 2, 3, and 4.

**Table 2:** Contingency between the different racial groups and birth outcomes

Races	Low birth weight	
	**0**	**1**
White	92%	8%
Black	84%	16%
AI/AN	90%	10%
Asian	90%	10%

**Fig. 5:**
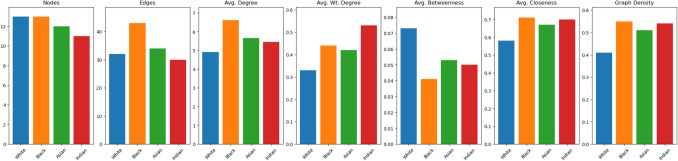
Network analysis results

Table 3 provides numerical comparisons of key network metrics across racial groups, while Fig. 5 visually represents these differences, allowing for clearer pattern recognition of comorbidity structures and disease connectivity.

**Table 3:** Network analysis results for different racial groups

Metrics	White	Black	Asian	AI/AN
Nodes (diseases)	13	13	12	11
Edges (comorbidities)	32	43	34	30
Number of clusters	2	3	3	3
Avg. degree (degree of a disease is the number of diseases directly connected to it)	4.92	6.61	5.66	5.45
Avg. wt. degree (degree calculated as a weighted sum of the strength of the comorbidities)	0.33	0.44	0.42	0.53
Avg. betweenness (number of times a disease is a bridge between pairs of diseases)	0.073	0.041	0.053	0.05
Avg. closeness (closeness centrality of a disease would represent how close a disease is to all the other diseases in the network)	0.58	0.71	0.67	0.70
Graph density	0.41	0.55	0.51	0.54

#### Network Properties

**Fig. 6:**
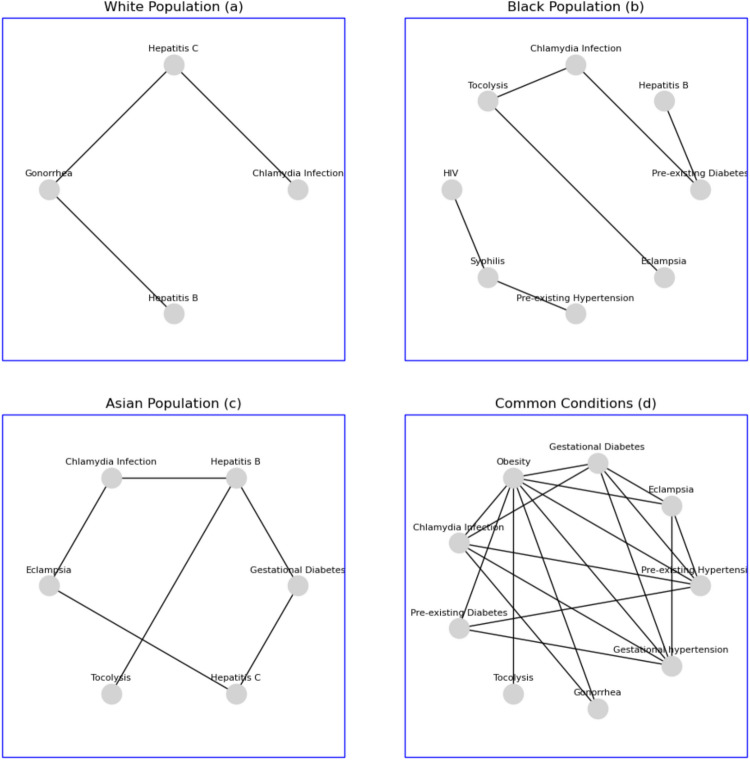
Exclusive comorbidities among individuals of different races

Network analysis revealed several key differences in comorbidity patterns across racial groups. All groups presented with a similar number of diseases (nodes), with the highest being shared by the White and Black groups (13 diseases each), followed by the Asian group (12 diseases) and AI/AN group (11 diseases). Although all 13 diseases were documented in both White and Black populations, no instances of HIV were recorded for the Asian cohort, and no cases of hepatitis C or syphilis were documented among AI/AN mothers. In terms of comorbidity connections (edges), the Black group exhibited the greatest number (43), which is substantially greater than that of the White (32), Asian (34), and AI/AN (30) groups. This suggests a more complex interconnectivity of diseases within the Black group’s comorbidity network. Our analysis revealed distinct comorbidity patterns among four racial groups: White, Black, Asian, and AI/AN. These unique comorbidities, observed exclusively within each racial group, are shown in Fig. 6.

White population:

- Chlamydia infection and hepatitis C infection
- Gonorrhea and hepatitis C
- Gonorrhea and hepatitis B

Black population.

- Preexisting diabetes and hepatitis B
- Chlamydia infection and tocolysis
- HIV and syphilis
- Chlamydia infection and preexisting diabetes
- Preexisting hypertension and syphilis
- Eclampsia and tocolysis

Asian population:

- Gestational diabetes and hepatitis B
- Chlamydia infection and eclampsia
- Hepatitis B and tocolysis
- Eclampsia and hepatitis C
- Chlamydia infection and hepatitis B
- Gestational diabetes and hepatitis C

AI/AN population:

- No unique comorbidities were identified in the AI/AN population within the scope of this analysis.

In the analysis of disease co-occurrence, the White population exhibited two distinct clusters, whereas the Black, AI/AN, and Asian populations each exhibited three clusters. Black women can be clustered into three groups. The first group includes STIs, such as gonorrhea, chlamydia, and syphilis, which raises the possibility of a behavioral risk factor overlap or an overlap in the transmission channels. Hepatitis C, B, and HIV are bloodborne diseases included in the second cluster. Preexisting diabetes, existing hypertension, gestational diabetes, gestational hypertension, obesity, and eclampsia are among the illnesses that have been linked together by the third cluster, suggesting that these conditions are related to underlying metabolic dysfunctions and cardiovascular hazards. The first cluster of White women comprises STIs and bloodborne illnesses (HIV, gonorrhea, hepatitis B/C, syphilis, and chlamydia), emphasizing the need for integrated approaches to blood safety and sexual health. The second cluster in White mothers includes hypertension, obesity, eclampsia, and diabetes, highlighting their interconnectedness within metabolic and hypertensive disorders Three clusters are also observed in Asian women. The first reflects the pattern of metabolic and pregnancy-related complications observed in the other populations, suggesting that these disorders are of general concern. The second cluster may indicate a possible association between serious pregnancy problems and infectious disorders because it pairs eclampsia with hepatitis B and C. The third cluster highlights sexual health as a major area of concern by combining STIs. Additionally, there are three clusters of AI/AN women. The first cluster may represent a complex interaction between the incidence of viral diseases and long-term health issues because it mixes chronic conditions (preexisting diabetes and hypertension) with infectious diseases (HIV, hepatitis B). The second, which focuses on STIs, suggests that sexual health is a crucial aspect of overall health. The third highlights the global reach of these health issues by capturing a range of metabolic and pregnancy-related problems, similar to trends observed in other racial groups. When considering the average number of diseases directly connected to each disease (avg. degree), the Black group again had the highest average (6.61) compared with the White (4.92), Asian (5.66), and AI/AN (5.45) groups. This indicates that, in the Black group, diseases tend to co-occur with a greater number of other diseases. The average weighted degree, which accounts for the strength of comorbidities, was highest in the AI/AN group (0.53). Despite having fewer diseases and connections, the comorbidities present in the AI/AN group were, on average, more significant than those in other groups, including the White (0.33), Black (0.44), and Asian (0.42) groups. The average betweenness centrality, indicative of a disease’s role as a bridge within the network, was highest in the White group (0.073), suggesting that diseases in this group may play a more central role in connecting other diseases. The Black group had the lowest average betweenness (0.041). Closeness centrality, which reflects the average proximity of disease to all other diseases in the network, was highest in the Black group (0.71), indicating a more tightly interconnected network, with diseases being closer to each other on average than in the White (0.58), Asian (0.67), and AI/AN (0.70) groups. Graph density, which is a measure of the overall connectivity of the network, was again highest in the Black group (0.55), indicating a higher level of overall interconnectivity among diseases in this group. The White group had the lowest graph density (0.41), suggesting a less dense comorbidity network.

### Network Analysis of Low Birth Weight and Normal Weight

Table 4 presents the prevalence of various conditions among mothers who gave birth to NBW and LBW infants. The percentages indicate the proportion of individuals diagnosed with each condition within each birth weight category. Statistical significance, determined using *p* values, highlights differences in prevalence between the two groups. The chi-square test was used to assess statistical significance, with *p* values provided in the final column.

**Table 4:** Chi-square analysis of the number of diseases and LBW

Feature	NBW (0)	LBW (1)	*p* value
Preexisting diabetes	1.0%	1.97%	< 0.001
Gestational diabetes	5.2%	5.07%	0.20
Preexisting hypertension	2.9%	6.7%	< 0.001
Hepatitis B	0.1%	0.15%	0.18
Hepatitis C	0.3%	0.59%	< 0.001
Gonorrhea	0.4%	0.69%	< 0.001
Syphilis	0.1%	0.20%	< 0.001
Chlamydia	2.2%	2.92%	< 0.001
Tocolysis	0.2%	0.62%	< 0.001
Eclampsia	0.6%	3.69%	< 0.001
HIV	0.1%	0.22%	< 0.001
Gestational hypertension	8.0%	18.43%	< 0.001
Obesity	33.8%	35.29%	< 0.001

**Fig. 7:**
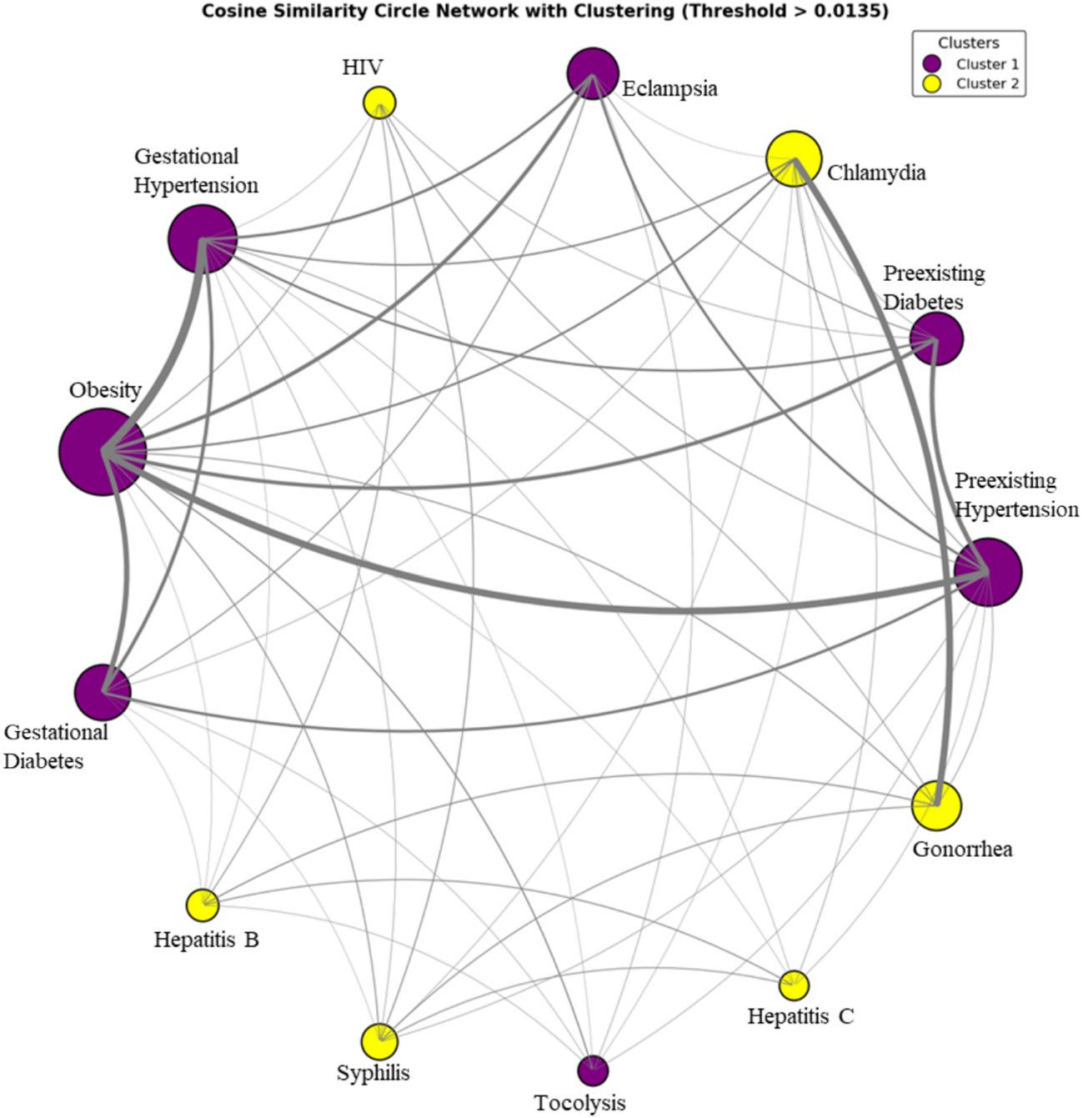
Network analysis of LBW

**Fig. 8:**
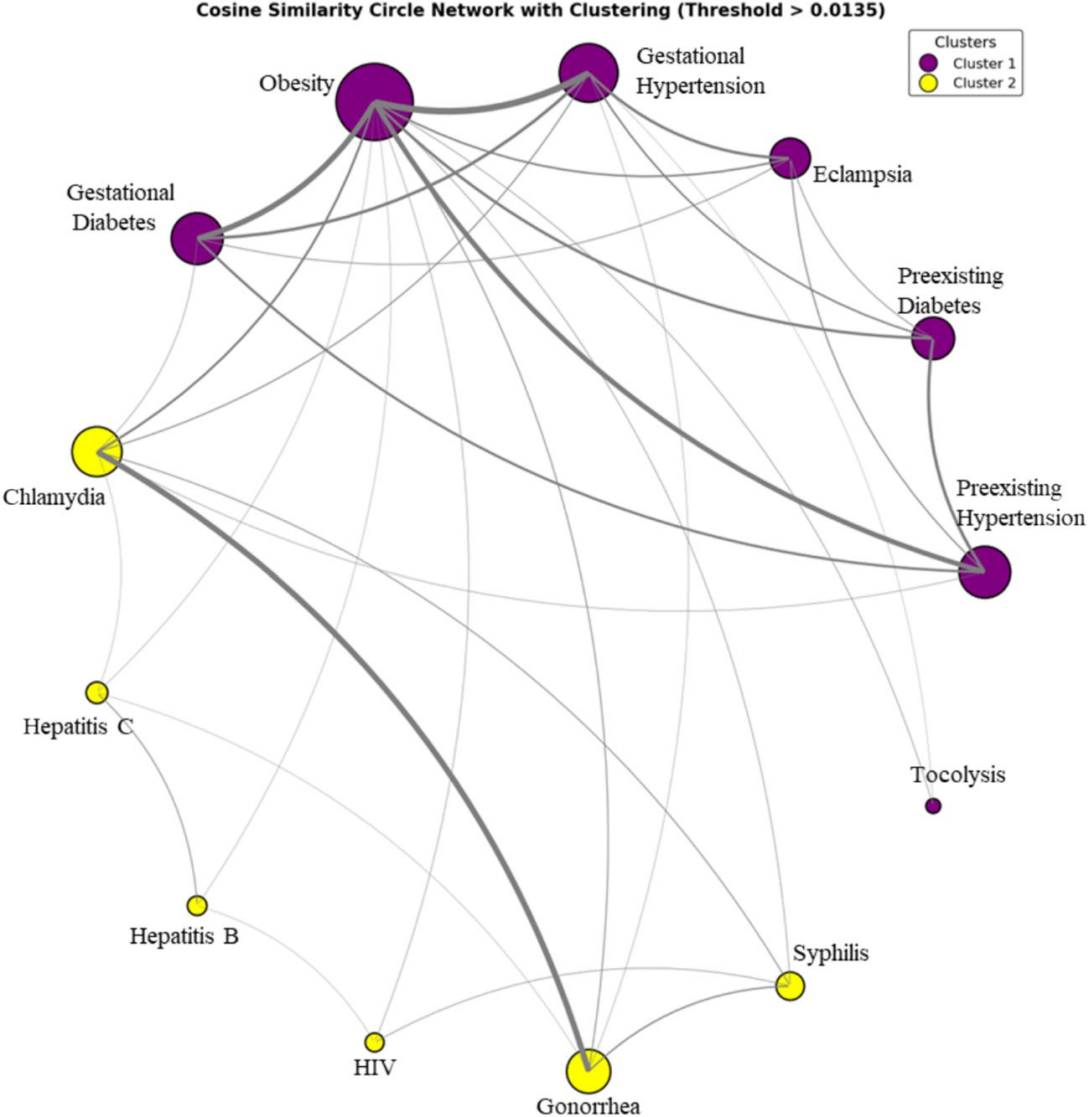
Network analysis of NBW

In Figs. 7 and 8, we demonstrate how LBW varies based on the presence of different diseases. To illustrate these differences, network analysis will be applied, highlighting the intricate relationships between the prevalence of multiple diseases and LBW outcomes.

Table 5 presents a comparison of network analysis results between the LBW and NBW groups. because of the nonnormality of the data, the Wilcoxon test, a nonparametric method, was employed to determine the statistical significance of the differences between the two networks.

**Table 5:** Network analysis results of the two groups

Metrics	Weight < 2500 g (low birth weight)	Weight ≥ 2500 g	Wilcoxon (normal—LBW)
No. of patients	47,261	396,641	NA
Nodes (diseases)	13 (0)	13	NA
Number of clusters	2	2	NA
Edges (comorbidities)	52 (+ 19)	33	NA
Avg. degree (degree of a disease is the number of diseases directly connected to it)	8 (+ 2.93)	5.07	0.002*
Avg. wt. degree (degree calculated as a weighted sum of the strength of the comorbidities)	0.51 (+ 0.17)	0.34	< 0.001*
Avg. betweenness (number of times a disease is a bridge between pairs of diseases)	0.03 (− 0.022)	0.052	0.30
Avg. closeness (closeness centrality of a disease would represent how close a disease is to all the other diseases in the network)	0.76 (+ 0.11)	0.65	0.034*
Graph density	0.66 (+ 0.24)	0.42	NA

*p value < 0.05

There were 47,342 patients in the LBW group and 396,641 patients in the NBW group. Both groups had 13 disease-related genes represented as nodes in the network.

**Fig. 9:**
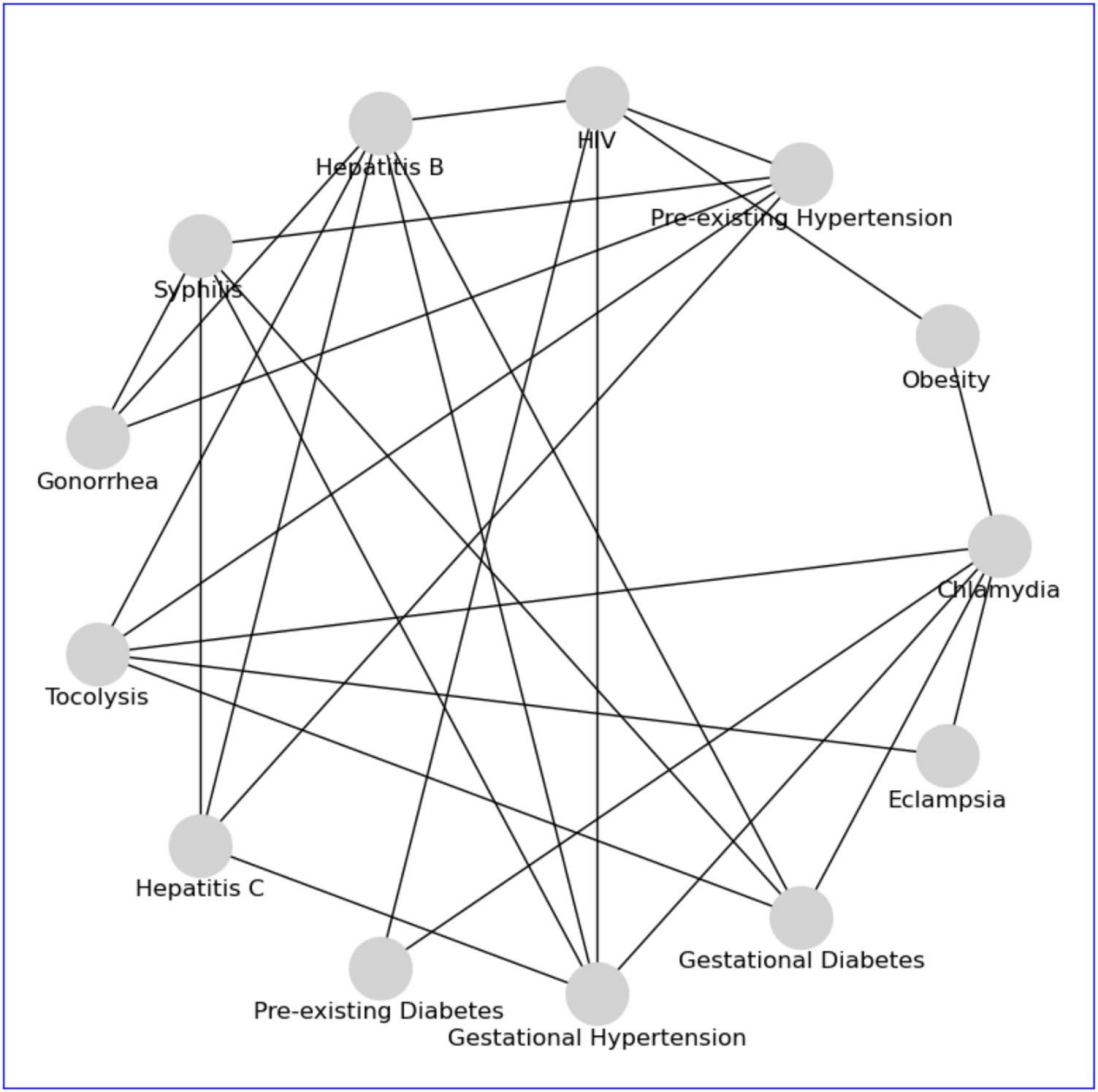
Exclusive comorbidities that exist only in LBW networks

There were 52 edges (comorbidities) in the LBW group, with a significant change indicated by “ + 19.” The NBW group has 33 edges. This finding suggests that there were more comorbidities in the LBW group. Figure 9 shows the comorbidities that existed exclusively in the LBW network.

In addition, the network analysis for both the LBW and NBW groups revealed two primary clusters that were consistent across both groups. The first cluster comprises conditions such as HIV, chlamydia, hepatitis B and C, syphilis, and gonorrhea. These may represent sexually transmitted infections or those commonly screened for during pregnancy. The second cluster included gestational diabetes and hypertension, preexisting diabetes and hypertension, obesity, eclampsia, and tocolysis, which are generally associated with pregnancy complications. This clustering pattern underscores shared pathways or risk factors among the conditions within each cluster.

The average degree in the LBW group was 8 (+ 2.93), which was greater than that in the NBW group (average 5.07; *p* = 0.002). This indicates that, on average, diseases in the LBW network are connected to other diseases.

The average weighted degree for the LBW group was 0.51 (+ 0.17), compared with 0.34 for the NBW group (p < 0.001). This means that, considering the weight of connections, diseases in the LBW network are more interconnected.

The average closeness centrality was 0.76 (+ 0.11) in the LBW group and 0.65 in the normal weight group (*p* = 0.034). A higher value in the LBW group suggests that, on average, diseases are more closely related to all other diseases in the network.

The density of the network for the LBW group was 0.66 (+ 0.24), which was much greater than the 0.42 density of the normal weight group. A higher density indicates a greater number of edges in proportion to the number of nodes, suggesting a more interconnected network in the LBW group.

### The Effects of Race and Disease Simultaneously

**Fig. 10:**
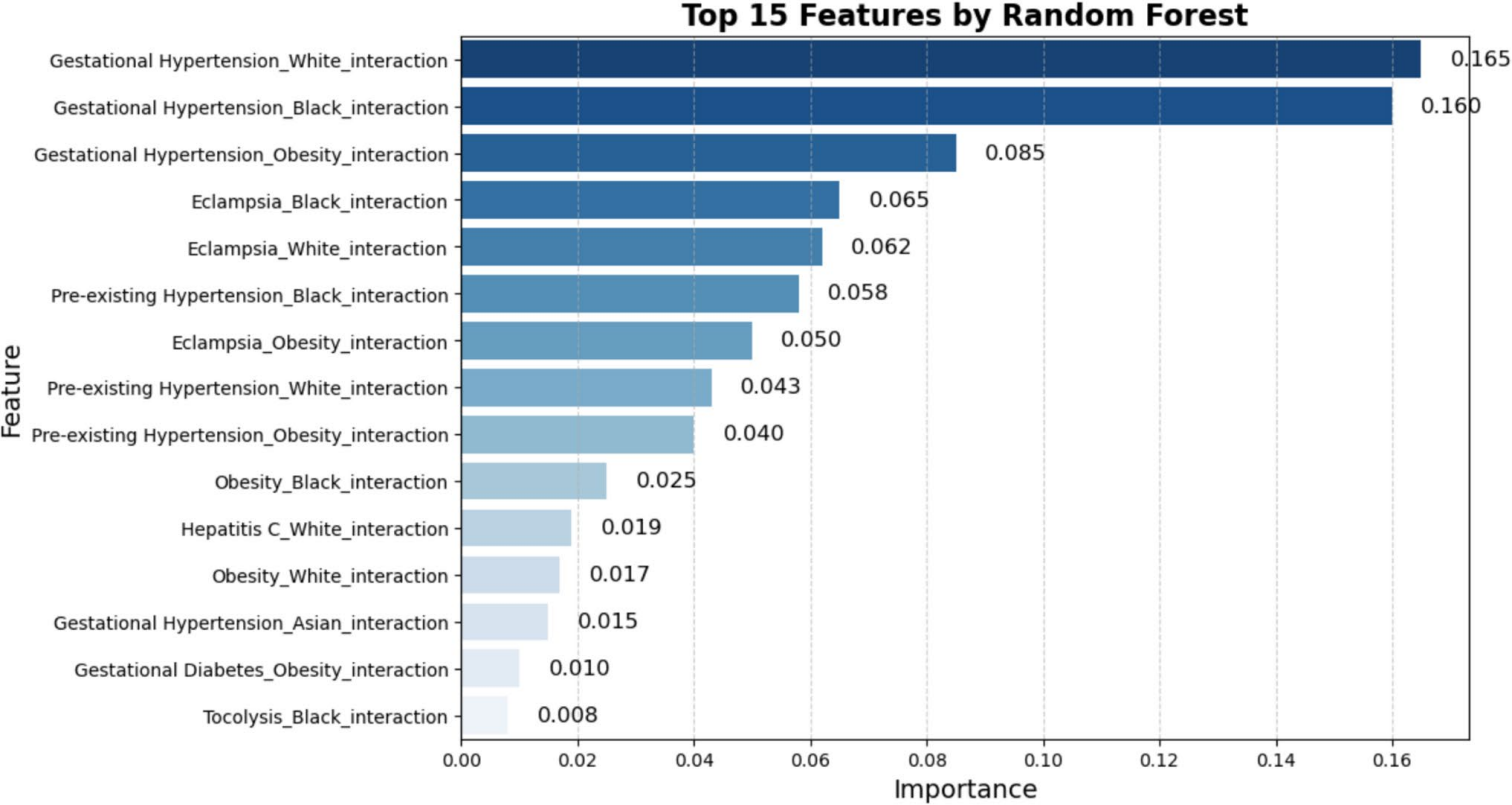
The most important features were identified by the random forest model

**Fig. 11:**
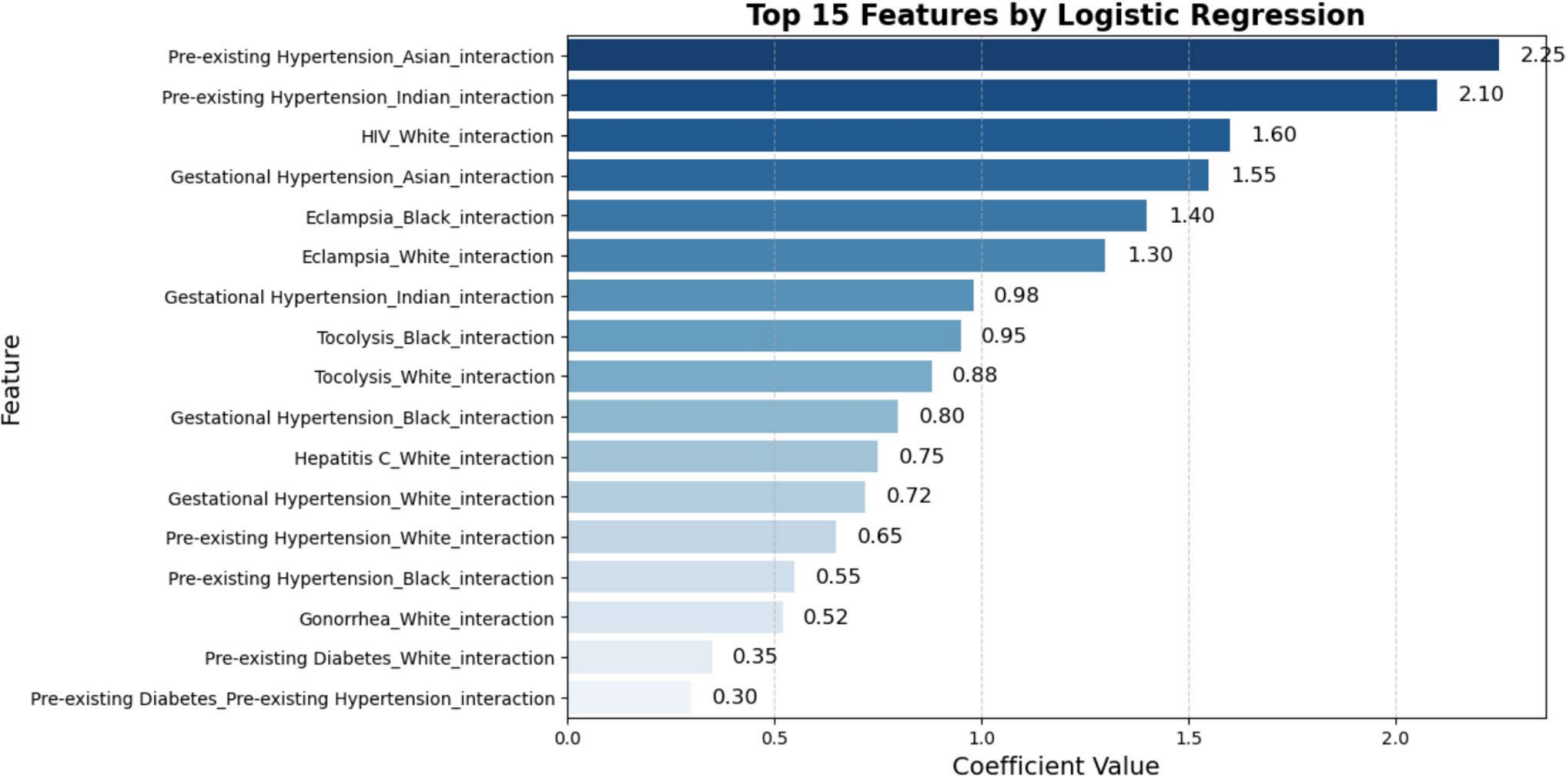
The most important features were identified by the logistic regression model

In this third section, the combined effects of race and disease on LBW were examined. Logistic regression, which is a linear model, is used to identify significant risk factors by the corresponding coefficients that measure the change in the log odds of LBW for a unit change in the predictor variable. On the other hand, random forest, which is a nonlinear model, assesses risk factors by evaluating their feature importance, which reflects how informative each variable is in splitting the data to improve model accuracy. Both algorithms provide different perspectives: Logistic regression offers direct relationships with the outcome, while random forest provides a measure of variable importance based on its contribution to the model’s predictive performance. Figures 10 and 11 visually present the results from these algorithms, allowing for a comparison of the risk factors identified by each method. The risk factors common to both methods will be considered for further discussion.

The common features between the logistic regression output and the random forest feature importance list are as follows:

- Gestational Hypertension_White_Interaction
- Gestational Hypertension_Black_interaction
- Gestational Hypertension_Asian_Interaction
- Eclampsia_White interaction
- Eclampsia_Black_interaction
- Preexisting Hypertension_White_interaction
- Preexisting hypertension_Black_interaction
- Hepatitis C_White_interaction

Gestational Hypertension_White/Black/Asian_Interaction: These features suggest that the risk of LBW associated with gestational hypertension may vary according to maternal race. The model indicated that this condition combined with the mother’s race could be a significant predictor of birth outcomes.

Eclampsia_White/Black_Interaction: Eclampsia is a risk factor for the White and Black populations.

Preexisting Hypertension_White/Black_Interaction: These interactions might indicate that the historical context of hypertension before pregnancy influences the birth weight of White and Black mothers.

Hepatitis C_White_interaction: The findings here suggest that white mothers with hepatitis C are at increased risk of having infants with LBW.

## Discussion

### Different Racial Groups

The results that have shown variations in maternal and birth-related metrics across racial groups can be influenced by a variety of factors, including socioeconomic status, cultural norms, access to healthcare, and historical and systemic inequalities. For example, white mothers having the highest average age at birth and the highest coverage of private insurance can be attributed to a variety of factors. White individuals, on average, have higher socioeconomic status than other racial groups in many regions [45, 46]. Higher socioeconomic status is often associated with factors such as higher education levels, better job opportunities, and higher income, which can lead to delayed childbearing and greater access to private insurance [47–49]. In addition, in some cultures or communities, there may be greater emphasis on establishing a career or financial stability before having children, which could contribute to White mothers having children at an older age on average [48].

Disparities in insurance coverage may reflect differences in access to healthcare services [50, 51]. This might explain why White and Asian mothers may have higher rates of private insurance [52] because of factors such as income level, employment status, or employer-provided benefits [47, 49]. White and Asian individuals, on average, have higher household incomes than Black individuals [53]. Higher income levels increase the likelihood of individuals affording private health insurance, which often provides more comprehensive coverage and better access to healthcare services. In addition, White and Asian individuals may be more likely to have stable employment with access to employer-sponsored health insurance than Black individuals, who may be more likely to be unemployed or work in jobs that do not offer health insurance benefits [5]. White and Asian individuals may be more likely to work for employers who offer health insurance benefits, while Black individuals may be more likely to work for employers who do not offer such benefits. Additionally, historical and systemic factors, such as past discriminatory practices in healthcare and employment, may have contributed to disparities in access to healthcare and insurance coverage among different racial groups [54].

The analysis revealed higher marriage rates among Asian mothers. This can be attributed to the fact that, in Asian cultures, marriage is highly valued and that there may be strong cultural expectations to marry and start a family. These cultural norms can influence individuals’ decisions regarding marriage and family planning. Asian Americans, on average, have higher levels of education and income than other racial groups in the USA [55]. A higher socioeconomic status is often associated with higher marriage rates [48], as individuals may feel more financially secure and able to support a family. In addition, many Asian Americans come from immigrant backgrounds, where traditional family values and expectations regarding marriage may be more pronounced [56]. Immigrant communities often maintain strong ties to their cultural heritage, including values related to marriage and family. Religious beliefs can also play a role in marriage rates among Asian Americans. Many Asian Americans practice religions that place a strong emphasis on marriage and family, which can influence individuals’ decisions regarding marriage [57, 58].

Black mothers were found to have the lowest average age at delivery, the highest average number of diagnoses, and the highest Medicaid coverage. This can be attributed to various factors, including socioeconomic factors, access to healthcare, and systemic inequalities [59]. Blacks, on average, have a lower socioeconomic status than White and Asian Americans [60]. A lower socioeconomic status is associated with a greater prevalence of health conditions because of factors such as limited access to healthy food, higher levels of stress, limited access to healthcare, and limited access to preventive care and health education programs [49, 61]. As a result, they may be more likely to have undiagnosed or untreated health conditions, leading to a greater average number of diagnoses. Medicaid expansion under the Affordable Care Act (ACA) has helped increase coverage among low-income individuals, including many Blacks [62]. Medicaid provides access to essential healthcare services for those who may not otherwise be able to afford them [63].

The percentage of Asian patients with no morbidities was the highest, and the percentage with more than two morbidities was the lowest. This can be attributed to a combination of factors, such as genetic factors, lifestyle factors, and cultural factors, in addition to socioeconomic status and access to healthcare [49, 60, 64, 65]. Genetic factors can play a role in the prevalence of certain health conditions. Some studies have suggested that Asian populations may have genetic predispositions that lower their risk for certain diseases compared with other racial groups [66]. Diet and lifestyle choices can also impact health outcomes [67]. For example, Asian diets are often rich in vegetables, fish, and other healthy foods, which may contribute to better overall health and lower rates of certain diseases. Cultural factors, such as traditional health practices and beliefs, can also play a role in health outcomes [68]. Some Asian cultures prioritize holistic approaches to health, which may include practices that promote overall well-being and prevent the development of multiple morbidities.

The differences among race groups in the factors mentioned above, such as socioeconomic factors, access to healthcare, environmental factors, and systematic inequalities, could be the main cause of the higher rate of LBW among Black mothers. Preexisting health conditions, such as hypertension and diabetes, and limited access to healthcare are disparities in the quality of care, which may contribute to the increased prevalence of these conditions among Black mothers and are risk factors for LBW.

### Effect of Race and Diseases: Network Analysis

Network analysis revealed that obesity, preexisting hypertension and gestational hypertension are important predictors of LBW among all races, highlighting the complex interplay of various factors contributing to LBW. It is well documented in the literature that obesity is a well-established risk factor for LBW [69, 70]. It is associated with various maternal complications, such as gestational diabetes and preeclampsia, which can increase the risk of LBW [71]. Obesity can also affect fetal growth and development, leading to LBW [72]. Preexisting hypertension, such as preeclampsia, which is a significant risk factor for LBW, increases the risk of complications during pregnancy. Hypertension can affect the placenta’s ability to deliver oxygen and nutrients to the fetus, resulting in LBW [73]. Gestational hypertension, or high blood pressure that develops during pregnancy, is associated with an increased risk of LBW because it can lead to reduced blood flow to the placenta, affecting fetal growth and development [74].

The network analysis suggested that these factors may interact with each other and with other factors to influence the risk of LBW. For example, obesity may increase the risk of developing hypertension during pregnancy, which, in turn, increases the risk of LBW. The identification of obesity, preexisting hypertension, and gestational hypertension as important predictors of LBW among all races has implications for intervention strategies. Efforts to reduce the prevalence of obesity and hypertension, as well as the effective management of these conditions during pregnancy, can help reduce the risk of LBW.

The present study has revealed differences in the incidence of gestational hypertension among racial and ethnic groups, aligning with previous research [75] that has documented disparities in disease prevalence. If gestational hypertension is more commonly diagnosed among White, Black, and Asian individuals than among AI/ANs in this study population, it may be a significant risk factor for LBW in these groups [76, 77]. However, differences in diagnosis rates could reflect variations in healthcare access, screening practices, and reporting rather than true prevalence disparities [78]. Structural inequities, including disparities in healthcare access, quality of prenatal care, and exposure to chronic stressors, may contribute to differential health outcomes.

Similarly, this study found that eclampsia and preexisting hypertension significantly increase the risk of LBW among White and Black individuals but not among AI/AN and Asian individuals [79]. Differences in diagnosis, treatment access, and underlying socioeconomic and environmental conditions may shape these patterns. The effect of these conditions on LBW may vary across racial and ethnic groups due to healthcare disparities, prenatal care quality, and broader social determinants of health. Addressing these inequities is essential for improving maternal and infant health outcomes across all populations.

The findings suggesting that white mothers with hepatitis C are at increased risk of having LBW could be the result of more recent and larger studies that have provided more robust evidence of this association [80, 81]. The impact of hepatitis C on LBW may vary among different populations or racial groups. Previous studies may not have accounted for this variability because there may have been limited previous research examining the impact of hepatitis C on LBW, leading to a lack of awareness about its potential effects. As our understanding of the impact of hepatitis C on pregnancy outcomes continues to evolve, it is important to consider this factor in prenatal care and interventions aimed at reducing LBW.

## Limitations

### Sample Size and Statistical Power

The present study may have had a larger sample size for White and Black individuals compared with other racial groups, providing more statistical power to detect significant associations between eclampsia, preexisting hypertension, and LBW in these groups. Smaller sample sizes for other racial groups could result in a lack of statistical significance for these factors.

### Different Types of Diseases

The data included only 13 specific diseases, hence restricting the analysis of these diseases. This limitation may prevent the exploration of associations between LBW and other potentially relevant health conditions not included in the dataset.

To overcome these limitations, the team is actively seeking to acquire data from the University of Alabama at Birmingham (UAB). This new dataset is anticipated to be more advantageous because it includes the ICD- 10 disease classification, which could offer a more balanced representation of different races and a broader range of diseases. By utilizing these more comprehensive data, the current study could help enhance its statistical power and improve the reliability of its findings across various racial groups and health conditions.

## Conclusion

Maternal deaths and negative outcomes, such as LBW, are largely preventable with adequate prenatal care, proper nutritional support, and education on healthy pregnancy practices. Regular prenatal visits allow healthcare providers to monitor both the mother’s and the fetus’s health, identify, and manage complications early, and provide guidance on nutrition and lifestyle choices. Adequate nutrition during pregnancy is crucial for fetal development and can help prevent LBW. Education on healthy pregnancy practices, including avoiding harmful substances and engaging in regular exercise, can further reduce the risk of negative outcomes. Addressing social determinants of health, such as access to healthcare, socioeconomic status, and environmental factors, is also essential for preventing maternal deaths and improving birth outcomes. By ensuring that all pregnant women have access to quality prenatal care and support, many of these adverse outcomes can be avoided, leading to healthier mothers and babies.

To the best of our knowledge, the current study is the first to consider the interaction between multiple factors, such as race and morbidity, and between morbidity and morbidity, and their impact on LBW to provide a better predictive statistical model that can improve screening detection rates. The study findings are as follows:

- Network metrics and analysis: Insights into the structure of the multimorbidity networks were provided through metrics such as degree, density, closeness, and betweenness centrality. These metrics aided in understanding the connectivity and influence of various diseases within the networks, informing more efficient healthcare strategies.
- Racial disparities in comorbidity networks: significant racial disparities were noted, with conditions such as preexisting hypertension and eclampsia being major risk factors for low birth weight (LBW) in the White and Black groups, but less so in Asian and AI/AN groups. This underscores the need for healthcare interventions tailored to the unique comorbidity profiles of each racial group.
- Disease influence on LBW and NBW: Separate network analyses for LBW and normal birth weight (NBW) revealed specific diseases that significantly reduce birth weight, pointing to the necessity of targeted medical interventions for pregnant women diagnosed with these conditions.
- Intersection of race and comorbidities: The use of feature selection methods like random forest and logistic regression helped clarify the complex interactions between race and comorbidities, demonstrating a significant impact on LBW outcomes. This indicates that both race and medical history should be considered in prenatal care and management.

## Data Availability

The code is not publicly available.

## Data Availability

The data is not publicly available.
